# Recent Progress in Direct Conversion of Methane to Methanol Over Copper-Exchanged Zeolites

**DOI:** 10.3389/fchem.2019.00514

**Published:** 2019-07-17

**Authors:** Min Bum Park, Eun Duck Park, Wha-Seung Ahn

**Affiliations:** ^1^Innovation Center for Chemical Engineering, Department of Energy and Chemical Engineering, Incheon National University, Incheon, South Korea; ^2^Department of Chemical Engineering and Department of Energy Systems Research, Ajou University, Suwon, South Korea; ^3^Department of Chemistry and Chemical Engineering, Inha University, Incheon, South Korea

**Keywords:** methane oxidation, methanol, zeolite, copper, process schemes

## Abstract

The conversion of methane into an easily transportable liquid fuel or chemicals has become a highly sought-after goal spurred by the increasing availability of cheap and abundant natural gas. While utilization of methane for the production of syngas and its subsequent conversion via an indirect route is typical, it is cost-intensive, and alternative direct conversion routes have been investigated actively. One of the most promising directions among these is the low-temperature partial oxidation of methane to methanol over a metal-loaded zeolite, which mimics facile enzymatic chemistry of methane oxidation. Thus mono-, bi-, and trinuclear oxide compounds of iron and copper stabilized on ZSM-5 or mordenite, which are structurally analogous to those found in methane monooxygenases, have demonstrated promising catalytic performances. The two major problems of theses metal-loaded zeolites are low yield to methanol and batch-like non-catalytic reaction systems challenging to extend to an industrial scale. In this mini-review, attention was given to the direct methane oxidation to methanol over copper-loaded zeolite systems. A brief introduction on the catalytic methane direct oxidation routes and current status of the applied metal-containing zeolites including the ones with copper ions are given. Next, by analyzing the extensive experimental and theoretical data available, the consensus among the researchers to achieve the target of high methanol yield is discussed in terms of zeolite topology, active species, and reaction parameters. Finally, the recent efforts on continuous methanol production from the direct methane oxidation aiming for an industrial process are summarized.

## Introduction

Natural gas will be a major energy resource in the transition period from the current petroleum-based energy economy to a renewable energy society in the future. Natural gas is presently used as a fuel for power generation or transportation, but often merely flared to the atmosphere without being utilized. Various attempts have been made to convert methane, which accounts for 70–90% of natural gas, to a more useful liquid fuel or chemicals (Periana et al., 1998; McFarland, 2012; Sushkevich et al., 2017). Thus, syngas is produced by steam reforming of methane (CH_4_ + H_2_O → CO + 3H_2_, Δ${\text{H}}_{298\text{K}}^{0}$ = +206.2 kJ mol^−1^), and this can be followed by either a Fischer-Tropsch process to hydrocarbons or methanol synthesis (CO + 2H_2_ → CH_3_OH, Δ${\text{H}}_{298\text{K}}^{0}$ = −90.7 kJ mol^−1^). However, this indirect route is highly energy consuming and also accompanied by multi-stage processes including a unit for water gas shift reaction (CO + H_2_O → CO_2_ + H_2_, Δ${\text{H}}_{298\text{K}}^{0}$ = −41.2 kJ mol^−1^). Therefore, it is strongly desirable to develop a low-cost, small-scale direct conversion process that can replace the indirect route.

Methane is a highly stable molecule difficult to activate due to its low electron and proton affinity, low polarity, high ionization energy, and strong C-H bond (~440 kJ mol^−1^) (Periana et al., 1998). The C-H bond of methane can be kinetically and thermodynamically activated by oxidation. However, the C-H bond (~47 kJ mol^−1^) in methanol which is one of the oxidative intermediates is weaker than that of methane, and thus completely oxidized to carbon dioxide under the reaction condition for methane activation. Therefore, it is desirable to develop an appropriate catalytic means to produce methanol selectively by direct methane oxidation. Methanol, incidentally, is a vital platform molecule to synthesize dimethyl ether, formaldehyde, light olefins, and even to gasoline through the methanol-to-gasoline (MTG) process (Tian et al., 2015; Yarulina et al., 2018).

**Figure 1:**
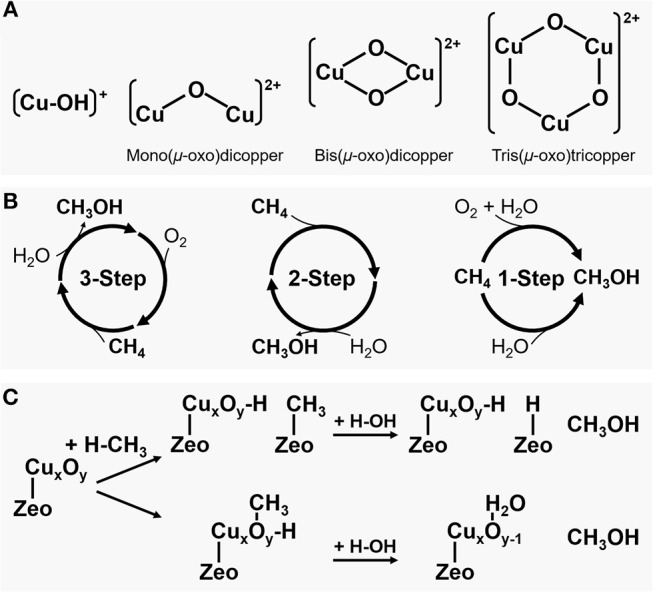
**(A)** Proposed copper active species formed inside the zeolite pores: (from left to right) a monovalent copper oxygen species attached to one zeolite framework Al, a divalent copper-oxo cluster forming one extra framework μ-oxo bridge attached to two zeolite framework Al, a divalent copper-oxo cluster forming two extra framework μ-oxo bridge attached to two zeolite framework Al, and a divalent copper-oxo cluster forming three extra framework μ-oxo bridge attached to two zeolite framework Al; **(B)** Schemes for direct methane oxidation to methanol; and **(C)** Two possible mechanisms of methanol production via partial oxidation of methane.

One promising pathway for direct conversion of methane to methanol is via partial oxidation of methane over a metal-containing zeolite catalyst (CH_4_ + 0.5O_2_ → CH_3_OH, Δ${\text{H}}_{298\text{K}}^{0}$ = −126.2 kJ mol^−1^), which mimics the methane oxidation by an enzyme (Kondratenko et al., 2017; Ravi et al., 2017; Tomkins et al., 2017; Dinh et al., 2018; Kulkarni et al., 2018; Mahyuddin et al., 2018a). As shown in Figure 1A, a mono-, bi- or tri-nuclear copper or iron complex similar to that of methane monooxygenases (MMOs) can be stabilized in the zeolite micropore structure such as ZSM-5 (framework type MFI) or mordenite (MOR) by ion-exchange of zeolite followed by successive activation with an oxidant (Snyder et al., 2018). The electrophilicity of the active metal oxide species allows methanol production by readily activating the strong C-H bond of methane even at a relatively low temperature. However, these metal-containing zeolite systems exhibit stoichiometric and non-catalytic reaction characteristics destitute of the continuous desorption of product such that a high methanol selectivity can only be achieved under the conditions of methane conversions <0.1% (Ravi et al., 2017; Dinh et al., 2018). These problems have to be resolved to extend the reaction to an industrial scale.

Although many studies on the direct conversion of methane over a zeolite containing transition metal ions of Fe, Co, Ni, Cu, and Zn and using N_2_O, H_2_O_2_, O_2_, and recently H_2_O as oxidants have been reported, the Cu-zeolite system with O_2_ or H_2_O has been regarded to be most promising for industrial application (Sushkevich et al., 2017; Lee et al., 2018). Cu-zeolites can be activated by both N_2_O and O_2_, unlike the Fe-zeolites on which methane activation sites cannot be formed with O_2_. The use of N_2_O and other oxidants (e.g., HNO_3_, H_2_SO_4_, NaClO, NaClO_2_, H_2_O_2_, etc.) cannot compete against freely available and environmentally friendly O_2_ and H_2_O. In this mini-review, therefore, the focus will be made on the direct methane oxidation to methanol on Cu-zeolite systems. After a short overview of this reaction system, the efforts made to obtain high methanol yields, and recent efforts for continuous methanol production are summarized.

## Multistep Methane Partial Oxidation Over Cu-Zeolites

Copper has been used as a catalyst for various oxidation reactions and, in particular, CuO is well-known as a catalyst for methane oxidation (Elwell et al., 2017). As shown in Figure 1B, the initially proposed direct methane to methanol conversion over a Cu-zeolite is carried out by a three-step cyclic process of oxygen activation, methane reaction, and methanol extraction (Ravi et al., 2017; Tomkins et al., 2017). In typical operation, Cu-zeolite is activated for several hours at near 450°C in an oxygen atmosphere and treated with an inert gas such as He to remove the O_2_ used in the activation of Cu-zeolite. Then methane is reacted for some time at about 200°C, and the produced methanol or methoxy group is desorbed or extracted from the Cu-zeolite using a solvent such as water to obtain methanol. A variety of Cu-zeolite catalysts have been evaluated during the last decade for such a multistep cyclic process, and the representative results are summarized in Table 1. An initial study using Cu-ZSM-5 showed a methanol yield of about 8.2 μmol ${\text{g}}_{\text{cat}}^{-1}$ with a methanol selectivity of over 98% with negligible carbon dioxide and carbon monoxide (Groothaert et al., 2005). More recently, much higher methanol yields were achieved, for example, ca. 125 and 169 μmol ${\text{g}}_{\text{cat}}^{-1}$ using Cu-SSZ-13 (CHA) and Cu-mordenite, respectively (Pappas et al., 2017, 2018; Dyballa et al., 2019). The methanol selectivity close to 100% was attained as a result of inert gas treatment between the oxygen activation and the methane reaction to prevent the excess oxidation of generated methanol in an O_2_-free atmosphere.

**Table 1:** Representative Cu-zeolite performances reported for the gas-phase direct methane oxidation to methanol^a^.

**Cu-zeolite**			**Oxidant**	**Temp.**, ^**°**^^**C**^		**Methanol yield**		**References**
**IZA code**	**Cu, wt%**	**Cu/Al**		**Activation**	**Reaction**	**$\mu {\text{mol g}}_{\text{cat}}^{-1}$**	**$\text{mol }{\text{mol}}_{\text{Cu}}^{-1}$**	
MFI	4.0	-	O_2_	450	200	8.2	0.01	Groothaert et al., 2005^b^
MOR	4.3	0.4	O_2_	450	200	13	0.02	Alayon et al., 2012
MFI	1.9	-	NO	150	150	0.63	-	Sheppard et al., 2014
CHA	4.9	0.4	O_2_	450	200	31	0.04	Wulfers et al., 2015
AEI	2.5	0.3	O_2_	450	200	36	0.09	Wulfers et al., 2015
AFX	5.0	0.3	O_2_	450	200	39	0.05	Wulfers et al., 2015
MOR	3.2	-	O_2_	450	200	160	0.32	Grundner et al., 2015
CHA	3.2	0.4	O_2_	450	200	45	0.09	Ipek and Lobo, 2016
CHA	3.2	0.4	N_2_O	450	200	35	0.07	Ipek and Lobo, 2016
MOR	3.0	-	O_2_	200	200	56	0.12	Tomkins et al., 2016^c^
MFI	-	0.3	O_2_	550	210	82	-	Narsimhan et al., 2016^d^
MFI	3.3	0.5	O_2_	450	200	89	0.17	Markovits et al., 2016
MOR	3.1	0.2	O_2_	450	200	30	0.06	Park et al., 2017^b^
CHA	4.5	0.8	O_2_	450	200	30	0.04	Park et al., 2017^b^
MAZ	6.0	0.3	O_2_	450	200	86	0.09	Park et al., 2017^b^
CHA	3.9	0.5	O_2_	500	200	125	0.20	Pappas et al., 2017
MOR	2.0	0.4	N_2_O	600	150	97	0.31	Kim et al., 2017
MOR	-	0.4	H_2_O	400	200	-	0.20	Sushkevich et al., 2017^e^
MOR	3.1	0.3	H_2_O	350	350	161	0.33	Lee et al., 2018^f^
MOR	2.3	0.2	O_2_	500	200	169	0.47	Pappas et al., 2018

a *All experiments were performed in a closed three-step cyclic process under atmospheric pressure that continuously extracts methanol by using on-line generated steam unless otherwise stated*.

b *Off-line methanol extraction with 1:1 water/acetonitrile mixture or water*.

c *Methane pressure of 37 bar*.

d *Methanol extraction by flowing a gas mixture comprised of 3.2 kPa of H_2_O, 0.0025 kPa O_2_, and balance CH_4_*.

e *Activation in He flow*.

f *Continuous one-step methanol production using 33% CH_4_ and 67% H_2_O*.

The methanol formed inside the zeolite pores can be extracted by two processes (Kulkarni et al., 2018). As shown in Figure 1C, one is a process in which methanol is produced by hydrolysis of a Brønsted methoxy group bonded to a zeolite framework oxygen atom with water. The other is a process in which the methyl radical of methane activated at the Cu-oxo site is directly adsorbed to a zeolite framework oxygen, and methanol is replaced by water used as a solvent. According to Kulkarni et al. who calculated the adsorption energies between methanol and different metal active species, it is difficult to spontaneously desorb the methanol molecules formed inside zeolite pores in most zeolite structures, but it is possible to desorb them using water. Theoretically, the adsorbed methanol could be desorbed with low water vapor pressure from almost any zeolite structures.

Although the structure of copper-oxo active species inside zeolite pores involved in the methanol production has been proposed since the beginning of the investigation, it remains controversial. The detailed spectroscopic and computational analyses for the active species are available in other reviews (Kulkarni et al., 2018; Snyder et al., 2018). Cu-mordenite, Cu-ZSM-5, and Cu-SSZ-13 have been studied extensively, and various active species such as monocopper (e.g., [CuOH]^+^) (Grundner et al., 2015, 2016; Li et al., 2016), dicopper (e.g., mono(μ-oxo)dicopper, [Cu_2_(μO)_2_]^2+^ and bis(μ-oxo)dicopper, [Cu_2_(μO)]^2+^) (Groothaert et al., 2005; Woertink et al., 2009; Tsai et al., 2014; Mahyuddin et al., 2018b,c), tricopper (e.g., tris(μ-oxo)tricopper, [Cu_3_(μO)_3_]^2+^) (Grundner et al., 2015; Markovits et al., 2016; Vogiatzis et al., 2017; Dandu et al., 2018; Mahyuddin et al., 2018b,c), and even sub-nanometer copper oxide clusters (Tomkins et al., 2017; Doan et al., 2018) have been proposed (Figure 1A). An early study of Cu-ZSM-5 showed a peak in the UV/Vis spectrum at 22,700 cm^−1^ corresponding to a bis(μ-oxo)dicopper site (Groothaert et al., 2005). On the other hand, using UV/Vis and Raman spectroscopies, the active species formed inside Cu-ZSM-5 pores was also claimed as a mono(μ-oxo)dicopper site (Woertink et al., 2009). The UV/Vis result for Cu-mordenite also detected the presence of the μ-oxo-dicopper site, but other active species were also proposed to be involved in methanol production (Alayon et al., 2012). Recently, EXAFS analysis of Cu-mordenite reported that a trinuclear copper-oxo cluster is the active species (Grundner et al., 2015; Markovits et al., 2016). For other zeolites such as Cu-beta (^*^BEA) and Cu-ferrierite (FER), despite only with a small amount of methanol formed, no active species like μ-oxo-copper clusters were observed (Smeets et al., 2005). These different results seem to indicate that there could be various active species depending on the zeolite structure, composition, and activation conditions.

## Methane Reactivity and Methanol Yield in Cu-Zeolite System

Various factors such as structure and composition of the zeolite used, the structure of copper active species, and reaction conditions should be considered to produce methanol selectively by direct partial oxidation of methane. Depending on the structure of the zeolite, different environment in which oxygen or methane is stabilized at the active copper species can be formed (Kulkarni et al., 2016; Zhao et al., 2016; Pappas et al., 2017; Liu et al., 2018). The C-H bond activation barrier energy of methane depending on the type of metal active species present in the zeolite pores was estimated (Kulkarni et al., 2018). The activation barrier energy decreased significantly among the transition metals from left to the right in the periodic table (Fe, Co, Ni, and Cu), and the energy by the μ-oxo-dicopper species was 107 kJ mol^−1^ lower than that by iron. Therefore, it was predicted that copper ion has a better reactivity to methane than the other transition metal ions. It was also claimed that activation barrier energy changes depending on the zeolite structures; the aluminum position and its bonding structure with metal cations affected the M-O-M angle, which influenced the activation barrier energy.

Recently, Cu-zeolites with small-pores such as SSZ-13, SSZ-16 (AFX), SSZ-39 (AEI), and SAPO-34 (CHA) were reported to exhibit better methane reactivity and methanol selectivity than the conventional Cu-ZSM-5 and Cu-mordenite (Wulfers et al., 2015; Ipek and Lobo, 2016; Kulkarni et al., 2016; Ipek et al., 2017; Pappas et al., 2017; Oord et al., 2018). These small-pore Cu-zeolites produced almost twice as much methanol per Cu-atom than the medium- and large-pore zeolites. It was reported that the activation energy necessary for breaking the C-H bond of methane, which is the rate determining step in the methane conversion, is controlled by the Cu-O-Cu angle, which was dependent on the crystallographic location in a zeolite structure and copper active species (Mahyuddin et al., 2017, 2018b). DFT calculations indicated that the activation energies for C-H bond dissociation by [Cu_2_(μO)2]^2+^ formed inside the small-pore zeolites (SSZ-13, SSZ-16, and SSZ-39) are lower than those for medium- (Cu-ZSM-5) and large-pore (Cu-mordenite) zeolites. Also, the 8- ring side pocket of mordenite zeolite was claimed to stabilize the catalytically active trinuclear copper-oxo clusters owing to the structural environment similar to that of the MMO (Grundner et al., 2015).

Park et al. prepared Cu-zeolites with 12 different structure types (i.e., MOR, EON, MAZ, MEI, BPH, FAU, LTL, MFI, HEU, FER, SZR, and CHA), and compared their methanol productivity by direct conversion of methane based on the copper content, activation temperature in an oxygen flow, zeolite structure, and zeolite precursor type (Park et al., 2017). The Cu-omega with MAZ structure showed the highest methanol yield among the zeolites. This was explained as a result of copper-oxo active species distributed in the three-dimensional 8-ring small-pore channel only available in the MAZ structure. *In-situ* UV/Vis analysis after catalyst activation under high temperature and oxygen atmosphere revealed that there exist various copper active species rather than a single copper active state. Four main factors contributing to obtain high methanol yield were suggested: (i) highly dispersed copper-oxo active species, (ii) copper active species formed in small-pore channels, (iii) appropriate level of activation temperature, and (iv) Cu^2+^ ion-exchanged from an H^+^-form zeolite. Cu-mordenite and Cu-omega catalysts prepared from their H^+^-forms produced a substantial amount of methanol even at 200°C both applied for oxygen activation and methane reaction. It was also reported that the Cu-mordenite prepared by liquid phase ion-exchange with its H^+^-form precursor showed the highest methanol yield compared to the other cation precursors (Dyballa et al., 2019). Furthermore, they suggested an optimum stoichiometry between Si, Al, and Cu of Cu-mordenite (i.e., Si/Al = 7 and Cu/Al = 0.18), which exhibited reproducible methanol productivity up to 169 μmol ${\text{g}}_{\text{cat}}^{-1}$.

## Continuous Methanol Production Over a Cu-zeolite System

At the beginning of the investigation using a Cu-zeolite system, an off-line extraction method was employed for methanol recovery. After the second step of methane reaction is completed, the reacted Cu-zeolite was recovered from the reactor, and the methanol was extracted by adding a solvent such as water. Subsequently, a closed multistep cyclic process that continuously extracts methanol by using on-line generated steam was proposed (Alayon et al., 2012). The cycling experiments showed successful regeneration of Cu-zeolite in a cyclic batch-wise operation; the catalyst deactivation can occur primarily due to sintering or leaching of Cu at high activation temperatures. The crucial problem of the multistep cyclic process was that these production steps proceed at different temperatures such that the first step of activating the copper in zeolite pores in an oxygen atmosphere is carried out at a high temperature of 450°C, and the contact with methane and extraction of methanol proceed at a relatively lower temperature of 200°C. This low temperature was necessary to prevent the methanol from excessively oxidized to CO or CO_2_. Consequently, an isothermal closed multistep looping system using Cu-ZSM-5, NO oxidizer, and on-line steam extraction was suggested, where NO activation, methane reaction, and methanol extraction were all carried out under the same temperature at 150°C, and a meaningful methanol yield of 0.63 μmol ${\text{g}}_{\text{cat}}^{-1}$ was obtained (Sheppard et al., 2014).

Meanwhile, methane conversion using Cu-mordenite and O_2_ at isothermal 200°C, but with increased methane pressure was also attempted (Tomkins et al., 2016, 2017); practically no methanol was obtained under the 200°C activation with methane at the atmospheric pressure but methanol yield rapidly increased as the methane pressure was increased, and about 56 μmol ${\text{g}}_{\text{cat}}^{-1}$ of methanol yield was achieved at 37 bar.

The methanol yield through the multistep cyclic looping process has been usually estimated and compared only by the amount of methanol produced per Cu-mol or weight of the catalyst. To develop a commercial methanol production process, however, the amount of catalyst and the total process time necessary to produce a meaningful amount of methanol should be considered. Recently, it was reported that a total 21 h was spent for one cycle of the three-step reaction over Cu-ZSM-5 including the temperature control of 210–550°C to produce about 82 μmol ${\text{g}}_{\text{cat}}^{-1}$ of methanol (Narsimhan et al., 2016). This result corresponds to approximately 4 μmol ${\text{g}}_{\text{cat}}^{-1}$ h^−1^ of continuous methanol production under the assumption that catalyst deactivation does not occur during the repeated cycles. It will be necessary to optimize the time required for each step of the stepwise cyclic process and to speed up significantly the methanol production over the entire cycle for its application to an industrial operation.

It has been also reported that under the same conditions using Cu-ZSM-5, simultaneous introduction of methane, oxygen, and steam continuously produced methanol in a steady-state with the production rate of 1.8 μmol ${\text{g}}_{\text{cat}}^{-1}$ h^−1^ methanol, which was about half the productivity obtained by the three-step process (Narsimhan et al., 2016; Dinh et al., 2018). Despite the co-presence of methane and oxygen, methanol was not excessively oxidized and was produced with high selectivity by keeping oxygen concentration to a very low level around 100 ppm. While meaningful, the very low methane conversion due to the use of excess methane and the potential over-oxidation of methanol by the co-presence of oxygen is still problematic.

Also, it has been reported that methanol can be continuously produced from the simultaneous introduction of methane and steam without any oxidant by the oxidizing ability of H_2_O (Sushkevich et al., 2017; Lee et al., 2018). The produced methanol was not excessively oxidized since steam has lower oxidizing power than oxygen. The catalytic cycle over Cu-mordenite under the co-presence of methane and steam at isothermal 350°C maintained stable methanol production for more than 500 min on stream, although the production rate of methanol was very low due to the thermodynamic limitation. For this reaction (CH_4_(g) + H_2_O(g) → CH_3_OH(g) + H_2_(g)), there is a thermodynamic restriction with Δ${\text{G}}_{298\text{K}}^{{}^{\circ}}$ ~ 117 kJ/mol, and the equilibrium methanol formation level is low.

## Conclusion and Outlook

In this mini-review, we examined the recent progress in methanol synthesis by direct methane oxidation over a Cu-zeolite catalyst. There have been various characterization and computational studies to identify the Cu-oxo active species involved in methanol production, and various types of mono-, di-, tricopper, and even sub-nanometer copper oxide clusters were proposed. Although there are several factors related to methane conversion, selection of a suitable zeolite is crucial to obtain the high methanol yield. It has been reported that Cu-zeolites with small-pores have shown the higher methanol yields than medium- and large-pore Cu-zeolites. The activation of methane C-H bond, which is considered to be the rate-determining step during the methane conversion, and Cu-oxo formed in the small-pores was calculated to have the lowest C-H bond activation energy. There has been considerable progress in continuous methanol production from direct methane conversion over Cu-zeolites, i.e., steady-state cyclic reaction with the simultaneous introduction of methane and steam with or without oxygen. However, considering the hourly production rate, the current status is just around one-hundredth of the methanol production rate via syngas from the commercially available indirect methane transformation. Therefore, it is necessary to both maximize the total number of copper active species in the zeolite catalyst and to reduce the time required for each cycle of the multistep process. There have been only limited attempts for isothermal oxidative activation and methane reaction, which can be an alternative but still needs a significant breakthrough for future implementation.

## Author Contributions

MP planned the contents and wrote the draft. EP did consulting and feedback on process data. W-SA initiated and supervised the work.

### Conflict of Interest Statement

The authors declare that the research was conducted in the absence of any commercial or financial relationships that could be construed as a potential conflict of interest.
